# A self-complementary AAV proviral plasmid that reduces cross-packaging and ITR promoter activity in AAV vector preparations

**DOI:** 10.1016/j.omtm.2024.101295

**Published:** 2024-06-26

**Authors:** Noah K. Taylor, Matthew J. Guggenbiller, Pranali P. Mistry, Oliver D. King, Scott Q. Harper

**Affiliations:** 1 Center for Gene Therapy, The Abigail Wexner Research Institute at Nationwide Children’s Hospital, Columbus, OH 43215, USA; 2 Department of Pediatrics, The Ohio State University College of Medicine, Columbus, OH 43210, USA; 3 Department of Neurology, University of Massachusetts Chan Medical School, Worcester, MA 01655, USA

**Keywords:** adeno-associated virus, AAV, AAV cross-packaging, AAV transfer plasmid, AAV biology, vector engineering, inverted terminal repeat, CTCF, insulators

## Abstract

Adeno-associated viral vectors (AAVs) are a leading delivery system for gene therapy in animal models and humans. With several Food and Drug Administration-approved AAV gene therapies on the market, issues related to vector manufacturing have become increasingly important. In this study, we focused on potentially toxic DNA contaminants that can arise from AAV proviral plasmids, the raw materials required for manufacturing recombinant AAV in eukaryotic cells. Typical AAV proviral plasmids are circular DNAs containing a therapeutic gene cassette flanked by natural AAV inverted terminal repeat (ITR) sequences, and a plasmid backbone carrying prokaryotic sequences required for plasmid replication and selection in bacteria. While the majority of AAV particles package the intended therapeutic payload, some capsids instead package the bacterial sequences located on the proviral plasmid backbone. Since ITR sequences also have promoter activity, potentially toxic bacterial open reading frames can be produced *in vivo*, thereby representing a safety risk. In this study, we describe a new AAV proviral plasmid for vector manufacturing that (1) significantly decreases cross-packaged bacterial sequences, (2) increases correctly packaged AAV payloads, and (3) blunts ITR-driven transcription of cross-packaged material to avoid expressing potentially toxic bacterial sequences. This system may help improve the safety of AAV vector products.

## Graphical abstract

**Figure:**
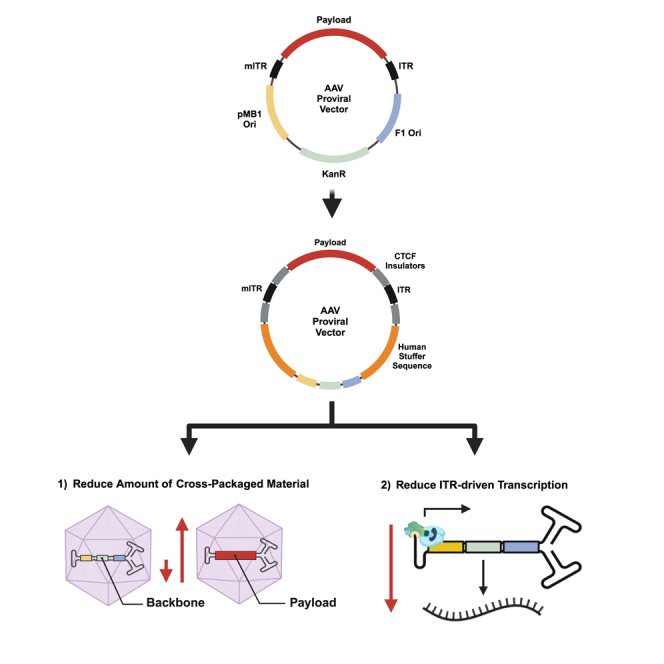

## Teaser

Scott Q. Harper and colleagues generated a novel, self-complementary AAV proviral plasmid that (1) decreases cross-packaging of the AAV transfer plasmid backbone, (2) blocks inverted terminal repeat-driven transcription, and (3) reduces packaging of potentially harmful bacterial DNA sequences by 70%.

## Introduction

After several decades of pre-clinical development, adeno-associated viral (AAV) vectors have emerged as promising clinical gene therapy delivery systems, with six Food and Drug Administration-approved therapies since 2017.^1^ With these products and additional prospective AAV-based therapies in the pipeline, issues related to vector manufacturing have become increasingly important. Natural AAV viruses encode two essential genes *(rep* and *cap*) encoding nine proteins, but cannot self-replicate, and require helper virus co-infection for replication.^2^ Recombinant AAV vectors are typically, but not exclusively, produced by co-transfecting HEK293 cells with three plasmids, separately containing: (1) AAV *rep/cap* genes, (2) adenovirus helper genes, and (3) a proviral plasmid containing AAV inverted terminal repeats (ITRs) flanking a therapeutic gene of interest (sometimes referred to as an AAV transfer plasmid). The ITR sequences define the AAV genome and contain AAV packaging signals. Thus, DNA sequences cloned between two AAV ITR sequences can be packaged within AAV capsids, with a maximum size of ∼4.7 kilobase pairs for single-stranded AAV (ssAAV), or ∼2.3 kilobase pairs if using a self-complementary, double-stranded genome (scAAV). For ssAAV, both ITRs are 145 base pairs (bp) in size, while scAAV genomes are created by deleting a portion of the terminal resolution sequence (aka Δtrs) in one ITR.^3^^,^^4^

Although typical research- and clinical-grade AAV vector preps largely contain correctly packaged genomes, essentially all also have non-uniform DNA payloads. From a recent review that cataloged genome purity of 30 AAV preps from 12 different studies, an average AAV vector prep contained about 3.5% packaged DNA contaminants, with a range from 0.5% to 13%.^5^ These unwanted nucleic acid contaminants are derived from manufacturing materials, including plasmids required for vector production. Thus, AAV contaminants can include portions of sequences used to propagate plasmids in bacteria, such as antibiotic resistance genes and bacterial sequences with high immunostimulatory CpG content and/or potential open reading frames (ORFs). In addition, AAV ITRs have promoter activity, and contaminating ORFs lacking their own promoters can still be transcribed from ITR promoters and translated into potentially deleterious proteins within the recipient.

Potential risks of mis-packaged bacterial sequences in AAV vectors were highlighted in a recent investigational new drug (IND)-enabling study in non-human primates, which demonstrated that even small amounts of AAV contaminants (referred to as cross-packaging or cross-packaged DNA) could be toxic to monkey brain.^6^ Specifically, 3 months after direct brain infusion of AAV1 vectors (three dose groups; 6 × 10^11^, 3 × 10^12^, and 1 × 10^13^ vector genomes [vg]), several animals developed ataxia, tremor, dysmetria, and brain lesions.^6^ Toxicity was dose-dependent and linked to ITR-driven expression of transcripts arising from cross-packaged AAV proviral plasmid backbone sequences and not the intended payload. Sequencing revealed that only 0.87% of the toxic vector preps contained cross-packaged proviral plasmid DNA, but even this small level of contamination (5.2 × 10^9^–8.7 × 10^10^ total vector genomes containing plasmid backbone sequences) triggered inflammation in monkey brains. These concerning results demonstrated that improved methods are needed to reduce contaminants, increase the percentage of correctly packaged AAV gene therapy products, and decrease ITR-driven transcription of cross-packaged material to avoid expressing potentially toxic bacterial sequences. Thus, the goal of this study was to improve the safety of AAV vectors by developing strategies to (1) dampen ITR-driven transcription of potentially toxic bacterial ORFs, (2) reduce cross-packaging of proviral plasmid backbone DNA in AAV vector preps, and thereby (3) increase the percentage of correctly packaged genomes.

## Results

**Figure 1:**
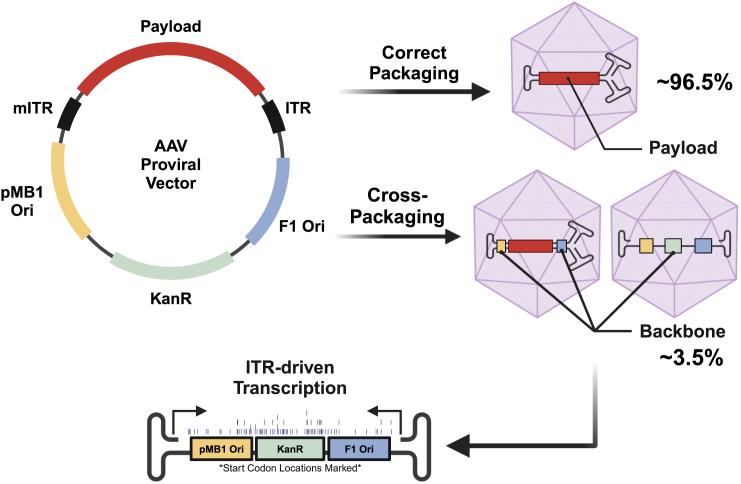
Schematic of ITR-mediated packaging of AAV proviral plasmids A proviral plasmid used to generate self-complementary AAV (scAAV) contains a therapeutic gene inserted between modified ITR (mITR) and full-length ITR sequences. In addition, the plasmid backbone contains typical sequences required for plasmid propagation and selection in bacteria (e.g., Ori, KanR). While the majority of AAV vectors package the intended payload, a small percentage may package the ITR-flanked plasmid backbone instead (cross-packaging). Average numbers from Brimble et al.^5^ Due to ITR-driven promoter activity, packaged bacterial or phage sequences can be transcribed and translated, producing potentially toxic products. Potential ATG start codons are noted as dashes.

Self-complementary AAVs (scAAVs) are produced by inserting therapeutic DNA payloads between a wild-type ITR and a modified ITR (mITR) containing a deletion in the terminal resolution sequence (Δtrs) (Figure 1). The base scAAV proviral plasmid our lab uses to generate scAAV vectors contains an ITR-flanked therapeutic payload (2,052 bp), inserted within a 3,523-bp plasmid backbone containing a kanamycin resistance gene (*KanR*) and bacterial and phage origins of replication (pMB1 *Ori* and F1 *Ori*) (Figure 1). Correctly packaged self-complementary AAV genomes maximally contain ∼2.3 kb of double-stranded DNA, including the ITR sequences. However, essentially all AAV preps also contain a small percentage of mis-packaged DNAs, in which ITR-flanked plasmid backbones, derived from the bacteria and bacteriophage sequences like those mentioned above, are inserted into AAV capsids instead of the intended payload (Figure 1). Since ITRs may have promoter or enhancer activity, these potentially toxic, cross-packaged prokaryotic or phage sequences can be transcribed and expressed in host cells, leading to safety concerns.^6^ We therefore hypothesized that AAV vector safety could be improved by reducing cross-packaging of bacteria- and phage-derived plasmid sequences in AAV preps, and interfering with ITR-driven transcription of any remaining cross-packaged material. Our approach to accomplishing these goals involved modifying our base scAAV plasmid in three fundamental ways: (1) flanking ITRs with insulator sequences to decouple ITR-driven transcription from adjacent ORFs; (2) inserting large, benign human intronic sequences in the plasmid backbone so that any cross-packaged material would more likely contain human, and not bacterial DNA^7^^,^^8^^,^^9^; (3) mutating all ATG start codons in the human intronic sequences to ensure that if any unintended ITR-driven transcripts were produced, protein translation could not be initiated.

### Insulators reduce transcriptional activity driven by wild-type and Δtrs-modified AAV2 ITRs in a scAAV proviral plasmid backbone

**Figure 2:**
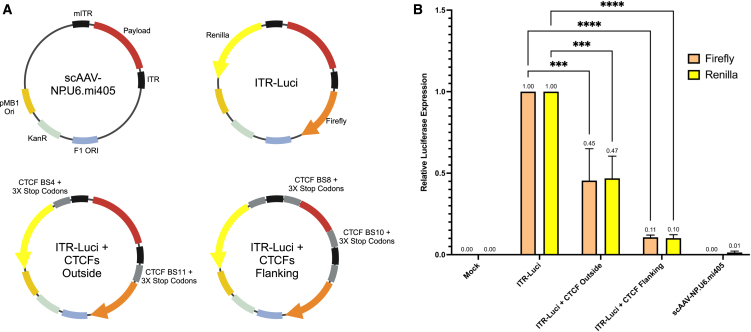
Full-length and mITR sequences exhibit promoter activity (A) Maps of C1 (scAAV-NP.U6.mi405), ITR-Luci, and ITR-Luci plasmids with indicated CTCF sites located in the plasmid backbone (Outside) or on both sides of the ITRs (Flanking). Payload refers to a U6.mi405 cassette containing a human collagen intron stuffer sequence. (B) Dual-luciferase assay measured from HEK293 cells transfected with the indicated plasmids, or a mock transfected control. The CTCF insulator sequences significantly reduced *Renilla* and firefly luciferase expression driven from the full-length and modified AAV2 ITRs. *N* = 3 individual experiments performed in triplicate. ∗∗∗*p* < 0.001, ∗∗∗∗*p* < 0.0001; two-way ANOVA with Dunnett’s multiple comparisons test. Error bars represent standard error of the mean.

Wild-type AAV2 ITRs possess promoter activity, which could enhance regulatory sequences present in correctly packaged genomes, or drive expression of potentially toxic bacterial sequences in mis-packaged genomes.^6^^,^^10^ We therefore hypothesized that AAV vector safety could be increased by interfering with ITR-driven transcription and attempted to accomplish this by flanking ITR regions with insulator sequences. To assess the possibility of decoupling ITR promoter activity from adjacent sequences, we first confirmed the transcriptional activation ability of each ITR in our AAV transfer plasmids, including from the Δtrs mITR (Figures 2A and S1). To do this, we modified an scAAV proviral plasmid containing a human collagen intron stuffer sequence and an engineered microRNA expression cassette designed to inhibit the *DUX4* gene (scAAV-NP.U6.mi405; Figure 2A).^11^^,^^12^^,^^13^^,^^14^ Specifically, we cloned a *Renilla* luciferase cDNA into scAAV-NP.U6.mi405 outside the mITR (i.e., in the plasmid backbone), and inserted a firefly luciferase cDNA outside the full-length, wild-type AAV2 ITR (we named this plasmid ITR-Luci) (Figures 2A and S1). Importantly, neither luciferase construct in ITR-Luci contained a promoter. We detected *Renilla* and firefly luciferase activity significantly above background (mock) 24 h after transfecting cells with ITR-Luci, or a positive control dual-luciferase plasmid that utilizes strong viral promoters to transcribe *Renilla* (SV40 promoter) and firefly (HSV-TK promoter) luciferase genes (psiCheck2; Promega; Figure S1). Although luciferase activity arising from psiCheck2 was ∼2–3 logs more robust than that produced from the ITR-Luci plasmid, these results confirmed the transactivation activity of AAV2 ITRs and demonstrated that the AAV2 ITR terminal resolution sequence (trs) is not required for promoter activity (Figure S1).

We next inserted two or four binding sites for the CCCTC-binding factor (*CTCF* gene) into the scAAV dual-luciferase (ITR-Luci) plasmid near the ITR sequences (Figure 2A and Table S1). CTCF is a highly conserved 11-zinc finger protein that binds specific DNA sequences (CTCF binding sites) and functions as an insulator.^15^ We hypothesized that inserting CTCF binding sites on both sides of the AAV ITRs could recruit endogenous CTCF proteins to flank ITRs and inhibit the transcription of adjacent ORFs. To do this, we inserted 80 bp of human genomic DNA containing previously characterized CTCF sequences present at the *HERC2*, *TBC1D2B*, and *ERCC4* loci, as well as one CTCF site derived from a long non-coding RNA (lncRNA) gene (ENSG000000260348) (Table S1).^16^ Specifically, we generated one plasmid with a CTCF binding site adjacent to each ITR inserted in the plasmid backbone (ITR-Luci + CTCF outside) and a second with CTCF binding sites flanking each ITR (ITR-Luci + CTCF flanking). Thus, the second plasmid contained four CTCF binding sites, with two in the plasmid backbone and two inside the AAV proviral genome (Figure 2A). We then assessed luciferase activity 24 h after transfection of these plasmids in HEK293 cells. Compared with the base scAAV dual-luciferase plasmid (ITR-Luci), insertion of CTCF binding sites in the outside position reduced both firefly and *Renilla* luciferase activity by about half (55% and 53%, respectively). Somewhat surprisingly, flanking both ITRs with CTCF sites (ITR-Luci + CTCF Flanking) further reduced firefly and *Renilla* luciferase activity by ∼90% (Figure 2). These results demonstrated that adjacent CTCF binding sites could interfere with ITR-driven transcription in an AAV proviral plasmid.

### Design of a novel scAAV vector backbone to reduce cross-packaging and decrease unwanted ITR promoter activity

**Figure 3:**
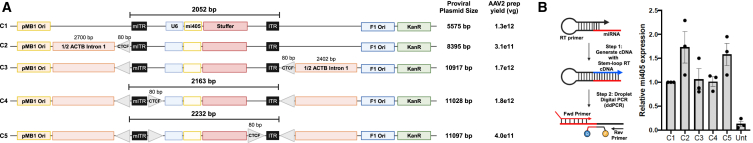
Generation of sequentially modified scAAV proviral plasmids and impacts on AAV2 titer and mi405 expression (A) The C1 construct represents our previously described scAAV.NP.U6.mi405 proviral plasmid. Indicated components were sequentially added to create plasmids C2, C3, C4, and C5 (details in methods). pMB1 Ori, a bacterial origin of replication. mITR, modified AAV2 inverted terminal repeat with a deletion in the terminal resolution sequence. U6 promoter and mi405 engineered microRNA targeting the *DUX4* gene linked to facioscapulohumeral muscular dystrophy (FSHD). Stuffer contains a portion of a human collagen intron. ITR, full-length AAV2 ITR. F1 Ori, phage origin of replication. KanR, kanamycin resistance gene. Human β-actin intron sequences (indicated) were inserted in the plasmid backbone on both sides of the AAV proviral insert. CTCF sites are indicated by gray arrowheads, with sequences listed in Table S1. Plasmid sizes and total AAV2 vector yields, determined by ITR-directed ddPCR assays, are shown. (B) HEK293 cells were transduced (10,000:1 multiplicity of infection [MOI]) with AAV2 preps generated with the indicated proviral plasmids (C1, C2, C3, C4, C5). A custom ddPCR assay was used to measure mature mi405 RNA levels. The plasmid backbone used to generate AAV had no significant impact on mi405 expression, compared with the parent C1 construct. (*N* = 3 independent experiments relative to mi405 levels produced by C1 for each experiment; mi405 expression levels from ddPCR normalized to human *RPL13A*. Repeated measures one-way ANOVA, *p* value = 0.4059). Unt = untransduced. Error bars represent standard error of the mean.

We next set out to incorporate CTCF binding sequences into a novel scAAV proviral plasmid designed to improve AAV vector manufacturing by reducing cross-packaging of plasmid backbone sequences (Figure 3A). Starting from our first-generation scAAV proviral plasmid (C1; aka scAAV-NP.U6.mi405), we sequentially inserted the same four, 80-bp human genomic sequences containing known CTCF binding sites as above (Figure 2 and Table S1). We also added to the plasmid backbone two large portions of the human β-actin intron 1 (ACTB-220, ENST00000675515.1), both of which exceeded the packaging capacity of a scAAV vector (Figure 3A). Finally, we mutated possible ATG start codons in each intron so that even if any cross-packaged material were to be produced and transcribed from ITR sequences, translational initiation of potential aberrant ORFs could not occur. We generated a total of five constructs (C1, C2, C3, C4, C5) and first tested the ability of each new plasmid to direct packaging of an identical payload in AAV2 vectors, which typically transduce cells *in vitro* more efficiently than other serotypes (Figure 3A). There were no significant differences in AAV2 vector yield among the five constructs, measured by a droplet digital PCR (ddPCR) assay targeting the ITR region (Figure 3A). Similarly, using a custom real-time PCR assay, we found no significant differences in mature mi405 expression in HEK293 cells transduced with each AAV2 vector prep (Figure 3B).

### Increased backbone size and addition of CTCF binding regions significantly reduce cross-packaging of AAV proviral plasmid backbone DNA

**Figure 4:**
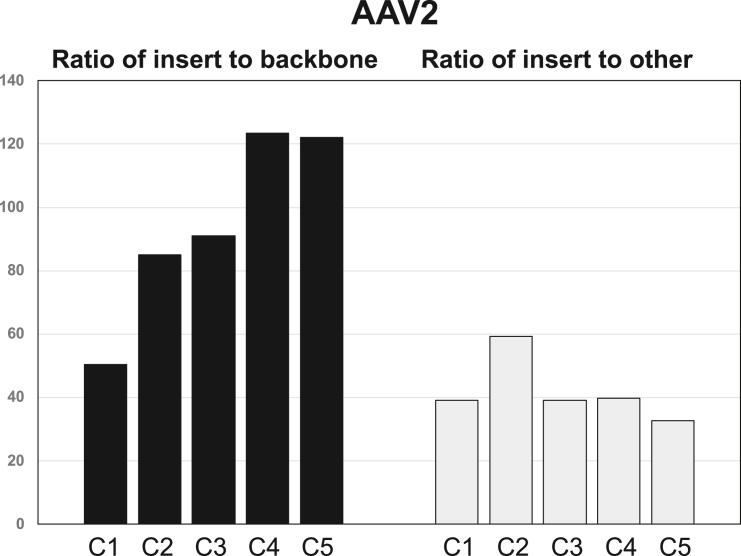
Optimized proviral plasmid backbones increase insert-to-backbone ratio in AAV2 vector preps Packaged AAV2 contents were identified using Illumina HiSeq short-read sequencing from AAV2 preps generated with the indicated proviral plasmids. Reads were mapped to the U6.mi405 insert and proviral backbone sequences, as well as “other” packaged DNA contaminants arising from the adenovirus pHELPER plasmid, pRep2/Cap2 plasmid, or HEK293 cell host DNA. The graph shows the ratio of mapped reads corresponding to the insert and backbone or insert and “other” DNA contaminants.

To assess the impact on cross-packaging of increasing plasmid backbone size with β-actin introns and CTCF binding sites, we began by sequencing the purified preps of the five sequentially modified constructs packaged into AAV2 capsids (C1, C2, C3, C4, C5) (Figure 3A and Table S2). We mapped sequence reads to either the AAV proviral plasmid (insert or backbone), or “other” DNase-resistant packaged sequences (corresponding to the Ad pHelper plasmid, AAV2 RepCap plasmid, and HEK293 cell human genomic DNA) (Table 1). Using the original proviral backbone (C1) to generate AAV2.U6.mi405 preps yielded 95.65% packaged insert and 1.9% proviral backbone sequence (Table 1). The percentage of cross-packaged AAV proviral backbone sequences reduced with each successive addition to the backbone (Figure 3A, Tables 1 and S2), while “other” sequences arising from AAV2 Rep/Cap plasmid, pHelper, and HEK293 DNA remained generally constant (mean 2.39% ± 0.21%) (Tables 1 and S2). The sequential reductions in packaged backbone DNA, when viewed another way, increased the ratio of insert to backbone DNA from 50:1 (C1) to 122:1 (C5) (Figure 4). In contrast, the ratio of insert to “other” DNA was similar in AAV2 preps made with C1 and C5, as well as the intermediate plasmids (Figure 4).

**Table 1:** NGS results of purified AAV2 vectors

Sample	Unique mapped reads	U6.mi405 insert	%	Proviral plasmid backbone	%	Other reads (Rep/Cap, pHelper, HEK293 cell DNA)	%
C1	79,646,796	76,186,037	95.65	1,510,889	1.90	1,949,870	2.45
C2	89,545,158	87,051,757	97.22	1,023,374	1.14	1,470,027	1.64
C3	115,235,278	111,161,460	96.46	1,221,212	1.06	2,852,606	2.48
C4	92,845,226	89,858,997	96.78	728,407	0.78	2,257,822	2.44
C5	86,751,679	83,499,317	96.25	684,081	0.79	2,568,281	2.96

Recombinant AAV2 preparations were generated from proviral plasmids C1 through C5. Next-generation sequencing of packaged genomes was performed on each prep using the Illumina HiSeq sequencing platform (2 × 150PE). Read counts are shown mapping to either the ITR-to-ITR insert containing our expected payload, the proviral plasmid backbone, or other sequences consisting of plasmids used in AAV production and the HEK293 host genome for which the vectors were produced in. While the percentage of “other” genomes remained relatively unchanged (mean 2.39% ± 0.21%, *p* < 0.05, t test) between preps C1 through C5, we observed both an increase in the percentage of the ITR-to-ITR U6.mi405 insert sequence, as well as a decrease in the percentage of packaged proviral plasmid backbone in C2–C5 compared with the original C1 plasmid.

**Figure 5:**
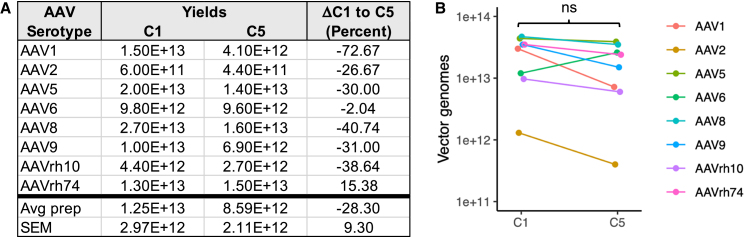
Total yield of 16 AAV vectors produced with C1 or C5 proviral plasmids (A) C1 or C5 proviral plasmids containing the U6.mi405/stuffer payload were used to generate eight different AAV serotypes, for a total of 16 AAV preps, as indicated. AAV2 data were already reported in Table S2 and Figure 4 but are used here for comparison. (B) Results plotted with line indicating the mean yield. Although there was a trend for lower vector yields using the C5 plasmid compared with C1, this difference was not significant (paired t test on log yields, *p* value = 0.080).

To confirm the results with AAV2, we generated 14 AAV preps from a panel of seven commonly used first-generation AAV serotypes and repeated DNA sequencing of purified vectors (Figure 5).^17^ We limited this round of sequencing analysis to the difference observed between AAV preps made with our original proviral plasmid (C1) and the final construct (C5). Except for AAV6, all preps generated with the C5 plasmid had lower yields than those produced with C1, as determined by ddPCR using an AAV2 ITR primer/probe set (Figure 5A). However, the overall difference in yields was not statistically significant (*p* = 0.080; paired t test on log of yields). The average prep using C1 plasmid was 2.68 × 10^13^ DNAse-resistant particles (DRPs) and 1.91 × 10^13^ DRP using C5 (Figure 5B). Even excluding AAV6, the decrease in average yields for C5 was modest and insignificant (2.89 × 10^13^ DRP using C1 vs. 1.81 × 10^13^ DRP using C5).

**Figure 6:**
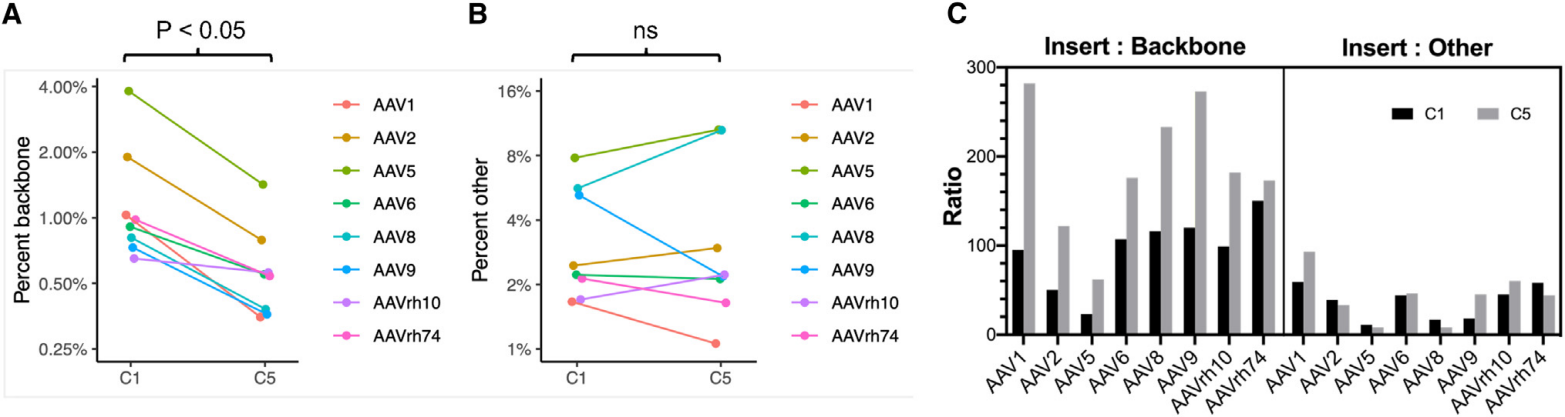
Optimized proviral plasmid backbones decrease cross-packaging and increase insert-to-backbone ratio in all AAV vectors tested (A) Percentage of cross-packaged backbone DNA in indicated U6.mi405/stuffer preps generated with proviral plasmid backbones C1 or C5. AAV preps Illumina HiSeq short-read sequencing was used to identify the DNA contents of the indicated AAV serotypes. The line indicates percent mean cross-packaged DNA and shows a significant decrease in cross-packaged material from the C5 backbone compared with C1 (*p* < 0.05, t test). (B) Percentage of “other” DNA contaminants packaged in indicated U6.mi405/stuffer preps generated with proviral plasmid backbones C1 or C5. Line indicates percent mean “other” DNA content within AAV preps. There were no significant differences in “other” DNA between AAV preps generated with C1 or C5 plasmids. (C) The graphs show that AAVs prepped with the C5 plasmid had a higher ratio of insert:backbone reads than corresponding vectors produced with the C1 plasmid. In contrast, the ratio of insert to “other” DNA was largely unchanged.

We again sequenced each AAV prep generated with plasmid C1 or C5 and found that every vector produced with C5 had reduced cross-packaged material compared with vectors generated with C1 (Figure 6A and Table S3). Vectors produced with C1 had on average 1.35% ± 0.38% cross-packaged material, compared with 0.62% ± 0.13% from those produced with C5, representing a statistically significant 46% reduction (*p* = 0.027, paired t test) (Figure 6A). In contrast, the percentage of “other” contaminants in C1- and C5- generated AAV preps were not significantly different (C1 mean, 3.60% ± 0.82%; C5 mean, 4.16% ± 1.41%; *p* = 0.386, t test) (Figure 6B and Table S2). Interestingly, both the C1- and C5-generated AAV8 preps showed higher levels of host cell DNA contaminants compared with the rest of the vectors tested (4.2% and 9.3%, respectively) (Table S3). Removing these values as outliers had little impact on the overall “other” DNA percentages (C1 mean minus AAV8, 3.31% ± 0.88%; C5 mean minus AAV8, 3.25% ± 1.24%) (Table S3). Similar to the AAV2 data in Figure 4, these results can be viewed another way to better appreciate the enhancement in the ratio of insert to backbone or insert to “other” DNA contaminants (Figure 6C). In particular, all serotypes showed improved ratio of insert to backbone, while the ratio of insert to “other” DNA contaminants did not markedly change (Figure 6C).

**Figure 7:**
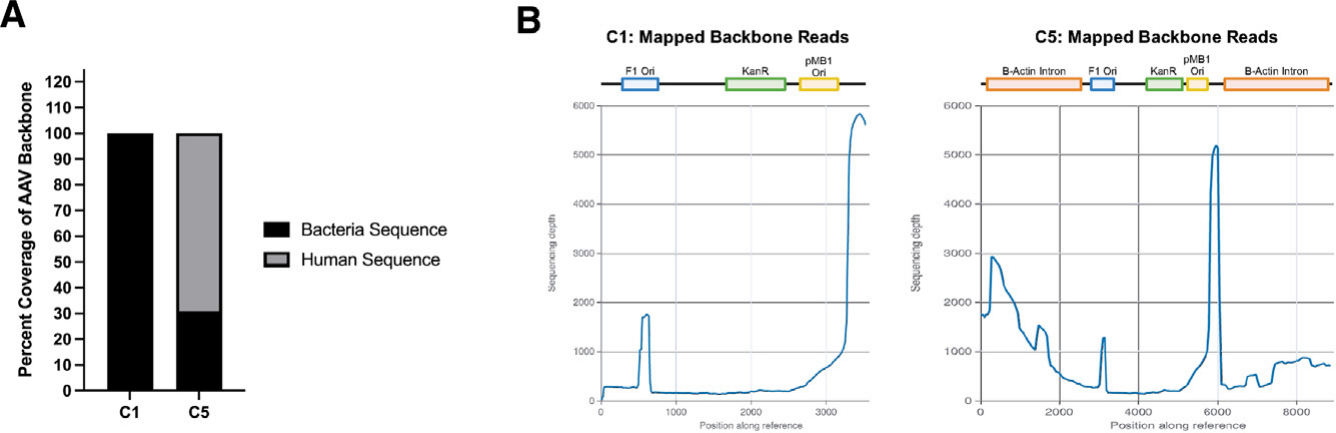
Optimized proviral plasmid backbones reduce bacterial DNA content present in packaged AAV vectors (A) Long-read sequencing demonstrated that AAV9 vectors packaged with the C5 proviral backbone had a 70% reduction in bacterial/phage DNA content, while those packaged with the original C1 plasmid contained 100% bacterial DNA. (B) The remaining 30% of prokaryotic/phage DNA present in the C5-generated AAV9 vector predominantly mapped to regions immediately adjacent to the β-actin introns.

Finally, we hypothesized that any remaining cross-packaged plasmid material arising from our C5 plasmids should predominantly contain human DNA sequences derived from the β-actin introns we inserted into the backbone. To determine this, we used long-read sequencing (PacBio) to identify the sequence configuration of the cross-packaged material from AAV9.U6.mi405 vectors prepped with C1 or C5 backbones. As expected, all cross-packaged material derived from the C1 plasmid was bacterial or phage in origin, while the cross-packaged genomes derived from C5 showed only 30% bacterial sequence, largely mapping to sequences immediately adjacent to the human β-actin introns (Figures 7A and 7B). In addition, we found small amounts of packaged human DNA, presumably derived from the host HEK293 cells we used to produce AAV vectors (Table S4). Finally, consistent with previous studies, we found that roughly half our scAAV.U6.mi405 genomes were truncated at two hot spots flanking the miRNA hairpin, effectively removing the U6 promoter from these genomes^18^^,^^19^ (Table S5, Figures S3 and S4). As a result, only ∼50% of genomes are capable of transcribing the mi405 product. In addition, we found slightly more truncations at the mi405 sequence in the AAV9 vector produced with C5 compared with C1 (28% vs. 23%; Table S5). In contrast, although long-read sequencing identified genome truncations throughout both C1- and C5-derived U6.mi405 inserts, most were very minor products (<1% of total reads) regardless of whether C1 or C5 was used for vector production. We noted a small number of truncations in the ITRs (5′ and 3′) occurring at frequencies above 1% (1.1%, 1.1%, 1.2%, 2.7%, 6.8%, 7.1% from C5), and the same truncations occurring at increased frequencies when produced from C1. Finally, we did not detect substantial numbers of truncations arising in the 180 bp of DNA added to the insert of the C5 clone (two 3X stop codons and two CTCF binding sites). For example, less than 1% of truncations occurred in CTCF8 and CTCF10 binding sites combined (CTCF8 truncations: 2,217/2,050,198 reads, 0.11%; CTCF10 truncations: 12,182/2,050/198 reads; 0.59%). This suggests that the additional sequences did not substantially impact vector titer.

Together, our results demonstrated that our new C5 AAV proviral plasmid significantly reduced cross-packaged material overall and that the remaining packaged backbone materials contained 70% human DNA.

## Discussion

To date, thousands of human patients have been safely dosed with AAV-based therapies, including several approved as clinical products. Although first-generation AAVs have a solid safety profile, as with almost any emerging modality, serious adverse events have occurred, and using the vector for gene therapy is not without risks. Some known safety concerns relate to high dose requirements for target tissue transduction, over-expression of the therapeutic payload, and immune responses to the capsids, gene products, or CpGs in the cargo DNA.^20^^,^^21^ In addition, the recent finding of toxicity in non-human primate brains related to ITR-driven expression of packaged DNA contaminants in AAV preps suggested imperfections in vector manufacturing may also be a cause for safety concerns, and inspired our group to initiate this study.^6^

In particular, we hypothesized that modifying the AAV proviral plasmid backbone used to manufacture AAV vectors could reduce the packaging and expression of potentially toxic DNA contaminants containing prokaryotic/phage sequences. Overall, we achieved the goals of this study by developing an AAV manufacturing system (C5) that produced the following: (1) a 46% reduction in packaged plasmid backbones; (2) improved ratio of packaged insert to backbone DNAs across eight serotypes; (3) 70% reduction of packaged bacterial sequences; and (4) blunting of ITR-driven gene expression; without significantly impacting (5) vector yield/titer; (6) expression of the therapeutic gene cassette (mi405); or (7) the ratio of insert to non-backbone DNA contaminants, including packaged host cell DNAs.

Despite these advances, additional work should be pursued to improve the system and better understand mechanisms. First, although we inserted four CTCF sites in the C5 plasmid backbone, resulting in ∼90% reduction of luciferase activity, only two would be present in linear AAV vectors with packaged insert or backbone (Figure 2). The C3 plasmid, which contains two CTCFs in the plasmid backbone and was created to mimic a cross-packaged genome, still significantly inhibited luciferase activity, but less robustly than C5 (Figure 2). These results suggest that CTCF proteins could bind cognate sites and sterically hinder or extrude AAV DNA away from the ITRs, ultimately blunting ITR-driven transactivation of luciferase genes. In future studies, we plan to determine if endogenous CTCF protein directly binds the CTCF sites in our AAV genomes or could possibly bind AAV plasmid DNAs during the manufacturing process, resulting in the trend toward decreased overall yield we observed from C5. In addition, we could assess the impact of CTCF sites on transcripts arising from cross-packaged sequences adjacent to ITRs in our U6.mi405 genomes.

Second, although we significantly reduced the percentage of cross-packaged backbone material in our AAV preps and replaced 70% of cross-packaged material with human β-actin intron DNA, the remaining 30% was still prokaryotic/phage in origin and largely derived from regions just adjacent to the human introns. We proposed that human DNA may be potentially more benign than bacterial DNA, and therefore hypothesize that we could further reduce the percentage of cross-packaged bacterial DNA simply by inserting larger human DNA sequences than the ones we used in this study. In addition, this system was designed for scAAVs, and single-stranded AAVs package longer genomes (although they contain the same amount of nucleic acid). Based on previous studies suggesting cross-packaging was facilitated by relatively smaller plasmid backbones (3.5 kb), it may be necessary to increase the β-actin (or similar) intronic sequences, so each arm exceeds the 4.7 kb packaging capacity of single-stranded AAV.^7^^,^^8^^,^^9^ Our data in this manuscript support this idea as well (Figures 4 and 6, and Table 1). Although the increased backbone size could theoretically impact vector yield, and we did see 5% more truncations at the mi405 sequence in C5-generated clones vs. those made with C1, we did not see substantial differences in vector production. The average AAV yield was slightly lower when using C5 compared with C1 (except AAV6), but overall yields produced by C1 and C5 were not significantly different (Figure 5). Moreover, in the AAV2 experiment, yields were essentially identical between vectors produced with the original C1 backbone compared with C3 and C4, despite the 2-fold greater size of the two latter constructs, and the slightly larger size of the inserts due to the addition of CTCF sites and stop codons.

Third, although we were primarily focused on reducing the packaging of the proviral plasmid backbone and increasing the ratio of packaged insert to backbone DNAs, we also noted that all our AAV preps contained similar amounts of pRep/Cap, pHELPER, and host cell DNAs. The backbones of the two helper plasmids contain some of the same sequences as those in our proviral plasmids, thereby potentially facilitating recombination. It may be possible to reduce packaged pRep/Cap and pHELPER sequences by creating unique plasmid backbones lacking homology with the others used in AAV manufacturing. Although the mechanisms by which host cell DNAs become packaged are unclear, this phenomenon may be unavoidable unless a cell-free AAV manufacturing system could be developed. In our study, the most abundant host cell reads mapped to the *COL2A1* intron we used as a stuffer sequence, which could represent the correct packaging of the insert but with genome deletions elsewhere (Table S4).

Finally, although theoretically our system should be safer than the first-generation plasmids currently used for AAV manufacturing, we do not yet know in practice if this is true. With large AAV doses administered in systemic gene therapies, even with the 42% reduction in cross-packaged material we reported here, patients would still receive massive amounts of contaminating DNA. For example, the total Zolgensma dose given to a 2-kg baby with spinal muscular atrophy (2.2 × 10^14^ genomes) would contain 3.0 × 10^12^ genomes of cross-packaged material if prepped with C1 (1.35%) or 1.4 × 10^12^ genomes using C5 (0.62%). However, by also reducing or eliminating the percentage of bacterial DNA present in the cross-packaged material, and blunting ITR-driven transcription, our system may indeed represent a safer alternative for AAV production. This could be most directly tested by repeating the Keiser et al. non-human primate study using AAV materials manufactured with our C1 and C5 plasmids. Regardless, we believe this study could represent an important step forward in improving AAV safety and serve as a foundation for further development of even more improved vector manufacturing systems.

## Materials and methods

### Generation of luciferase reporter constructs

The scAAV-NP.U6.mi405 sequence, which we called C1 in this study, was previously described.^11^^,^^12^ The ITR-Luci construct was generated by first cloning an AseI-flanked insert containing promoter-less *Renilla* luciferase and a synthetic poly-adenylation signal into the AseI site of C1 in the reverse orientation, adjacent to the AAV2 mITR. We then cloned a PvuI-flanked insert containing promoter-less firefly luciferase and a synthetic poly-adenylation signal into the PvuI site of C1+*Renilla* luciferase in the forward orientation, adjacent to the full-length AAV2 ITR. To generate the “ITR-Luci + CTCFs Outside” construct, we first cloned the CTCF binding site from the ENSG00000260348 lncRNA gene into a HindIII site located in the plasmid backbone adjacent to the mITR-Δtrs (CTCF4; Table S1), and then inserted the CTCF binding site from the *ERCC4* gene into the plasmid backbone adjacent to the full-length AAV2 ITR using SpeI (CTCF11; Table S1). Last, to generate the construct “ITR-Luci + CTCFs Flanking,” CTCF binding sites from the *HERC2* and *TBC1D2B* genes were added inside the AAV proviral genome, adjacent to the mITR and ITR sequences using BbsI and SacII cloning sites, respectively. All inserts were synthesized by Genscript Biotech (Piscataway, NJ).

### Cell culture, transfection, and dual-luciferase assay

HEK293 cells were cultured according to manufacturer’s instructions (ATCC, Manassas, VA). To perform the dual-luciferase assay, 65,000 cells were cultured in a white 96-well plate and transfected with indicated plasmids (Lipofectamine 2000, Invitrogen, Carlsbad, CA). Twenty-four hours later, media was removed and replaced with 30 μL of 1X Passive Lysis Buffer (Dual-Luciferase Reporter Assay, Promega, Madison, WI). Cells were vortexed on a plate shaker at 425 rpm for 20 min. Luminescence was then measured following the manufacturer’s instructions (Promega, Madison, WI) on a GloMax Discover machine (Promega, Madison, WI).

### Generation of novel AAV backbone constructs

Plasmids C2–C5 were sequentially produced beginning with the C1 base plasmid. Specifically, C1 was modified to create C2, which was then used to create C3, and so on for C4 and C5. To generate C2, we designed an AseI-flanked cassette containing 2,700 bp of the human β-actin (*ACTB*) partial intron 1 (ACTB-220, ENST00000675515.1) in which all potential ATG start codons were mutated to ATT. In addition, we added the CTCF binding site from the ENSG00000260348 lncRNA gene between the mITR and β-actin intron (Table S1, CTCF4 sequence).^16^ Finally, we added UAG stop codons between the mITR and CTCF4 sequences in all three reading frames. This insert was synthesized by GenScript (Piscataway, NJ) and cloned into the AseI site adjacent to the mITR-Δtrs. We used a similar strategy to generate C3, but the insert fragment was generated using an additional 2,402 bp of the human β-actin intron with ATGs mutated to ATT, the CTCF binding site from *ERCC4*, and UAG stop codons in all three reading frames (Table S1, CTCF11). This fragment was PvuI flanked and cloned into the PvuI site of C2, in the plasmid backbone adjacent to the full-length ITR. C4 was generated by cloning an AscI-flanked CTCF binding site from the *HERC2* gene, along with UAG stop codons in each reading frame into the AscI site of C3, adjacent to the mITR in the AAV proviral genome (Table S1, CTCF8). C5 was generated by cloning a BamHI/SacII-flanked CTCF binding site from the *TBC1D2B* gene, along with UAG stop codons in each reading frame into the BamHI/SacII sites of C4, adjacent to the full-length ITR inside the proviral genome (Table S1, CTCF10).

### AAV2 transduction and ddPCR measurement of mature mi405 expression

All AAV preps were generated by Andelyn Biosciences (Columbus, OH) using small-scale, adherent HEK293 cell transfections of pHELPER, pRep2Cap “X,” where “X” corresponds to specific capsids based on serotype, and the relevant proviral plasmids indicated in the results section. All vectors were purified by iodixanol gradient ultracentrifugation and FPLC purification. Titers and yields were determined by Andelyn Biosciences using AAV2 ITR primer/probes and ddPCR assay.

AAV2 vectors carrying C1, C2, C3, C4, or C5 were added at a 10,000 multiplicity of infection (MOI) to HEK293 cells (plated at 1,000,000 cells per well on six-well plates). Forty-eight hours later, total RNA was isolated using the *mir*Vana miRNA Isolation Kit, with phenol (Invitrogen, Carlsbad, CA), following the manufacturer’s instructions. One microgram of total RNA from each sample was treated with DNase I (Invitrogen, Carlsbad, CA) for 30 min at 37°C. Samples were then heat-inactivated by incubating at 75°C for 10 min cDNA was made using the High-Capacity cDNA Reverse Transcription Kit (Applied Biosystems, Foster City, CA) with both Oligo(dT)18 Primer (Thermo Scientific, Waltham, MA) and a custom miRNA RT primer to the mature mi405 sequence.^12^ All cDNAs were diluted to a 25 ng/μL final concentration. ddPCR was performed on the Bio-Rad (Hercules, CA) QX200 AutoDG ddPCR system according to the manufacturer’s protocol. To detect copies of mature mi405, forward primer (5′ CGGCCCAAACCAGATCTGAATC 3′), reverse primer (5′ GTGCAGGGTCCGAGGT 3′), TaqMan probe (5′ ATACGACGTCCAGGAT 3′), and cDNA final concentrations were set to 1.5 μM, 0.70 μM, 0.20 μM, and 0.5 ng, respectively. Droplets were quantified using Bio-Rad Quantasoft - QX Manager Software (version 2.0).

### AAV genome sequencing

C1 through C5 were packaged into AAV2, and additionally, constructs C1 and C5 were packaged into AAV serotypes 1, 5, 6, 8, 9, rh10, and rh74 by Andelyn Biosciences (Columbus, OH). At least 1 × 10^11^ vector genomes (vg) as DRPs were sent to Azenta Life Sciences (Burlington, MA), and AAV genome sequencing was performed using the Illumina HiSeq sequencing platform (2 × 150 PE). Reference sequences of individual constructs, relevant RepCap plasmids, and pHELPER plasmid sequences were provided to Azenta for read mapping. Reads not mapping to the provided reference sequences were mapped to the human reference genome (GRCh38 assembly). A concatenated reference sequence was generated for each sample. PacBio SMRT sequencing and read mapping were performed by Azenta for only AAV9-packaged C1 and C5. Pbmm2 (version 1.13.0) was used to align CCS reads for each sample to the corresponding concatenated reference sequence from before. Graphs of backbone coverage in Figure 7 were generated by EPI2ME Labs (Oxford Nanopore Technologies, Oxford, UK). Assignment of reads to genes was based on an overlap of at least 15 bp with genes (including introns) from GENCODE annotations (release 44), using the R GenomicRanges package.^22^

### Statistical analysis

AAV2 production yields and mi405 expression were compared with repeated measures one-way ANOVA. AAV production yields and percent of cross-packaged AAV between constructs 1 and 5 across all serotypes were compared with paired t tests, on log-transformed values where noted. Significance level cutoff for all comparisons was *p* < 0.05. Data are presented as mean ± SEM. Statistical analyses were performed using GraphPad Prism 9 Software (La Jolla, CA) and R.

## Data and code availability

All data are included in the manuscript or the supplemental information. GEO accession numbers for sequencing data will be provided upon request. Additional data requests may be made to the corresponding author.

## Supplemental information

Document S1. Figures S1–S4 and Tables S1, S2, and S4

Table S3. Next-generation sequencing (NGS) to identify packaged DNA contents of all first-generation AAV vectors used in the study AAV vectors carrying the same intended payload (U6.mi405/collagen intron stuffer) were treated with DNase before isolating packaged DNA within indicated AAV capsids. Purified AAV contents were then identified using Illumina HiSeq sequencing from AAV preps generated with the indicated proviral plasmids. Reads were mapped to the U6.mi405 insert and proviral backbone sequences, as well as “other” packaged DNA contaminants arising from the Ad pHELPER plasmid, pRep2/Cap”X” plasmids (where X = indicated serotype), or HEK293 cell host DNA.

Table S5. Identification of truncations and truncation hot spots in the U6.mi405 inserts produced using C1 or C5 backbones Relevant features are indicated.

Document S2. Article plus supplemental information
