# Supplementary material for: A self-complementary AAV proviral plasmid that reduces cross-packaging and ITR promoter activity in AAV vector preparations
Source: Mol Ther Methods Clin Dev. 2024 Jun 26;32(3):101295. doi: 10.1016/j.omtm.2024.101295 (PMC11320455; doi:10.1016/j.omtm.2024.101295)
Supplement: Document S1. Figures S1–S4 and Tables S1, S2, and S4 [file mmc1.pdf]

OMTM, Volume *32*

Supplemental information

A self-complementary AAV proviral plasmid that reduces cross-packaging and ITR promoter activity in AAV vector preparations

Noah K. Taylor, Matthew J. Guggenbiller, Pranali P. Mistry, Oliver D. King, and Scott Q. Harper

Table S1. Sequences of CTCF DNA-binding sites inserted into ITR-Luci and novel AAV backbone vectors

CTCF insulator sequences from Kim et al, “Analysis of the vertebrate insulator protein CTCF binding sites in the human genome”, Cell, 2007 Mar 23; 128(6): 1231-1245, Supplementary Table 7. The canonical CTCCC-binding motif is bolded and underlined in each sequence.

**CTCF4**<br>AAAGGGCCTGAAAGTCTCCACAATGACATG<u>**ATGGCCAGCAGAGGGCGCAT**</u>GGCCCTGGGGACCTCGACCCCTCTCCAGGG<br>11:69,909,184-69,910,994<br>lncRNA gene: ENSG00000260348
**CTCF8**<br>GGACCTTCGAGGTGATATATCTGTGACTCC<u>**TGGCCCCCTCTGCTGGACAT**</u>CCTGAGAGAAACAGCCCAATCGCCGGCCTT<br>15:28123590-28123669<br>*HERC2* gene: ENSG00000128731
**CTCF10**<br>CAGCTTGGCCATGGTTCTTGGGGCCTCCCA<u>**AGGCTGCCCCTGCTGGCAAA**</u>GGCATCCTGATGCACCCGCCCCAGGGTGAG<br>15:77994758-77994837<br>*TBC1D2B* gene: ENSG00000167202
**CTCF11**<br>AGACGTGAATGGAGCCTGATATCTGCAAGC<u>**TACACCGACAGGGGGCGCCA**</u>GCCCCTACAGGTGACTCCATGAATCTTCGG<br>16:13919818-13919897<br>*ERCC4* gene: ENSG00000175595

Table S2 Next generation sequencing (NGS) to identify packaged DNA contents of AAV2 vectors

AAV2 vectors carrying the same intended payload (U6.mi405/collagen intron stuffer) were treated with DNase before isolating packaged DNA within AAV2 capsids. Purified AAV contents were then identified using Illumina HiSeq sequencing from AAV2 preps generated with the indicated proviral plasmids. Reads were mapped to the U6.mi405 insert and proviral backbone sequences, as well as “other” packaged DNA contaminants arising from the Ad pHELPER plasmid, pRep2/Cap2 plasmid, or HEK293 cell host DNA.

NGS results of purified AAV2 vectors							
**Sample**	**Unique Mapped Reads**	**U6.mi405 insert**	**Insert %**	**Proviral Plasmid Backbone**	**Backbone %**	**Other Reads (Rep/Cap, pHELPER, HEK293 cell DNA)**	**Other %**
**AAV2 C1**	**79,646,796**	**76,186,037**	**95.65**	**1,510,889**	**1.9**	**1,949,870**	**2.45**
**AAV2 C2**	**89,545,158**	**87,051,757**	**97.22**	**1,023,374**	**1.14**	**1,470,027**	**1.64**
**AAV2 C3**	**115,235,278**	**111,161,460**	**96.46**	**1,221,212**	**1.06**	**2,852,606**	**2.48**
**AAV2 C4**	**92,845,226**	**89,858,997**	**96.78**	**728,407**	**0.78**	**2,257,822**	**2.43**
**AAV2 C5**	**86,751,679**	**83,499,317**	**96.25**	**684,081**	**0.79**	**2,568,281**	**2.96**

Table S3 Next-generation sequencing (NGS) to identify packaged DNA contents of all first-generation AAV vectors used in the study

Table included as separate Excel file. AAV vectors carrying the same intended payload (U6.mi405/collagen intron stuffer) were treated with DNase before isolating packaged DNA within indicated AAV capsids. Purified AAV contents were then identified using Illumina HiSeq sequencing from AAV preps generated with the indicated proviral plasmids. Reads were mapped to the U6.mi405 insert and proviral backbone sequences, as well as “other” packaged DNA contaminants arising from the Ad pHELPER plasmid, pRep2/Cap”X” plasmids (where X = indicated serotype), or HEK293 cell host DNA.

Table S4. Top 10 reads from host DNA sequences packaged in AAV9 vectors

Reads were identified from long-read sequencing of AAV9 vectors prepped with C1 or C5 proviral plasmid backbones. Packaged reads not mapping to insert, backbone, pHELPER, or Rep/Cap sequences were mapped to the human reference genome to characterize "host" cell DNA sequences and tabulated based on the genes (including introns) that they overlapped with by at least 15bp. The top hit in both preps was *COL2A1*, which is derived from the collagen intron stuffer sequence in our U6.mi405 payload, but the other sequences appear to be randomly inserted into AAV genomes.

C1			C5		
**Gene**	**Chromosome**	**# Reads**	**Gene**	**Chromosome**	**# Reads**
COL2A1	chr12	17	COL2A1	chr12	23
ENSG00000255872	chr9	5	ATG5	chr6	5
ESRRG	chr1	4	PRDM16	chr1	4
CASZ1	chr1	3	HDAC4	chr2	4
RUNX2	chr6	3	ZMIZ1	chr10	4
RASA4B	chr7	3	DANT2	chrX	4
CYRIB	chr8	3	CAMTA1	chr1	3
PRKG1	chr10	3	ATP2B2	chr3	3
SEPTIN9	chr17	3	PALLD	chr4	3
SIPA1L3	chr19	3	GRID2	chr4	3

Table S5. Identification of truncations and truncation hot-spots in the U6.mi405 inserts produced using C1 or C5 backbones. Table included as separate Excel file. Relevant features are indicated.

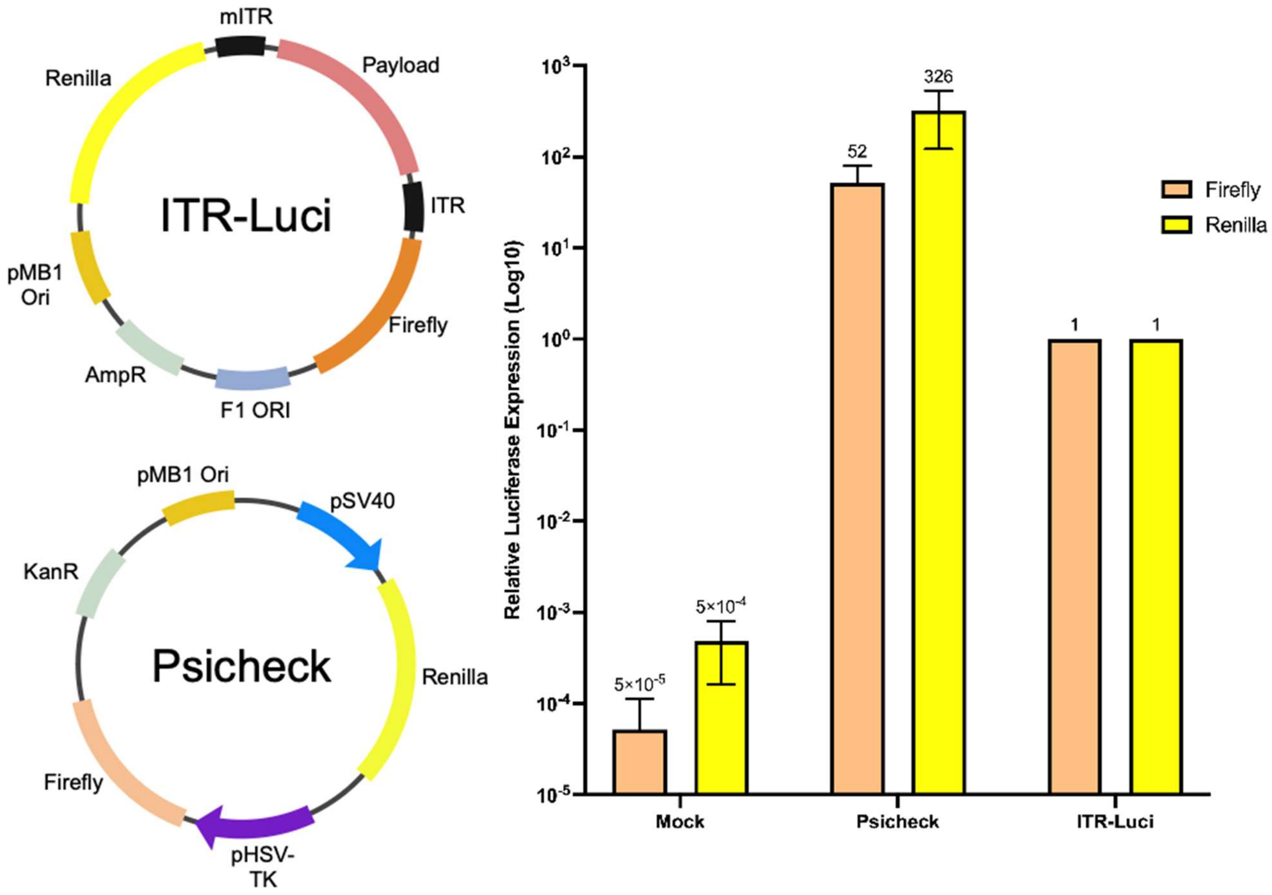

Fig S1. Assessment of ITR-driven luciferase activity compared to a dual luciferase plasmid utilizing strong viral promoters

ITR-Luci plasmid contains promoterless *Renilla* and Firefly luciferase genes cloned into the plasmid backbone adjacent to mITR and ITR sequences, respectively. Psicheck2 is a positive control plasmid utilizing strong viral promoters (SV40 and HSV-TK) to drive *Renilla* and Firefly luciferase expression, respectively. Luciferase activity was assessed 24 hours after plasmid transfection in HEK293 cells, compared to mock transfected HEK293s. ITR-driven Renilla and Firefly luciferase activity was significantly increased above mock, but less than that achieved by viral promoters. N=3 replicates performed in triplicate. $P < 0.05$; two-way ANOVA with Dunnett's multiple comparisons test.

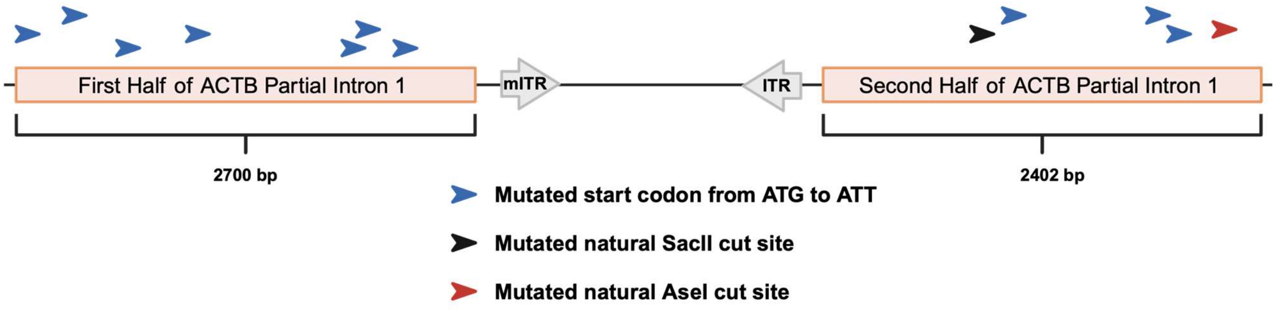

Fig S2. Locations of mutated sites along the ACTB intron sequences

ATG start codons along both halves of the ACTB intron inserted into the proviral backbone. Blue arrows mark the locations where these start codons originally presided before they were mutated from ATG to ATT. Additionally, natural SacII and AseI sites were mutated in the second half of the partial ACTB intron 1 for cloning purposes (marked as black and red arrows).

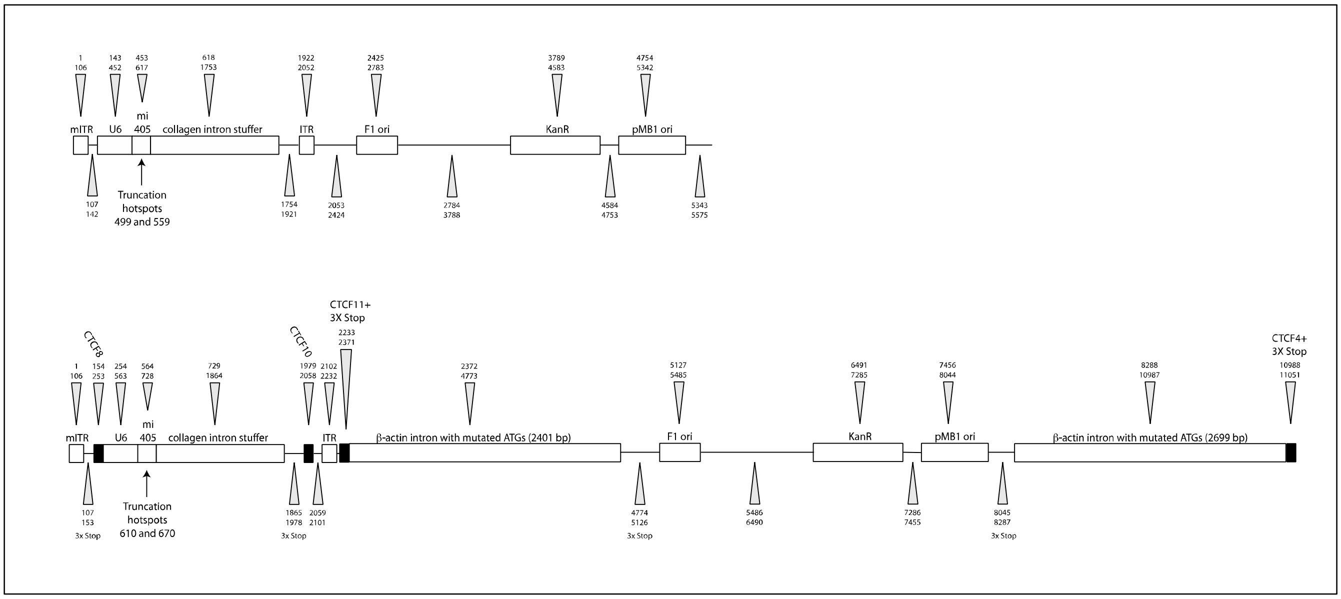

Fig S3. Map of U6.mi405 inserts with C1 or C5 backbones.

Relevant features are indicated. Triangles with numbers indicate nucleotide positions.

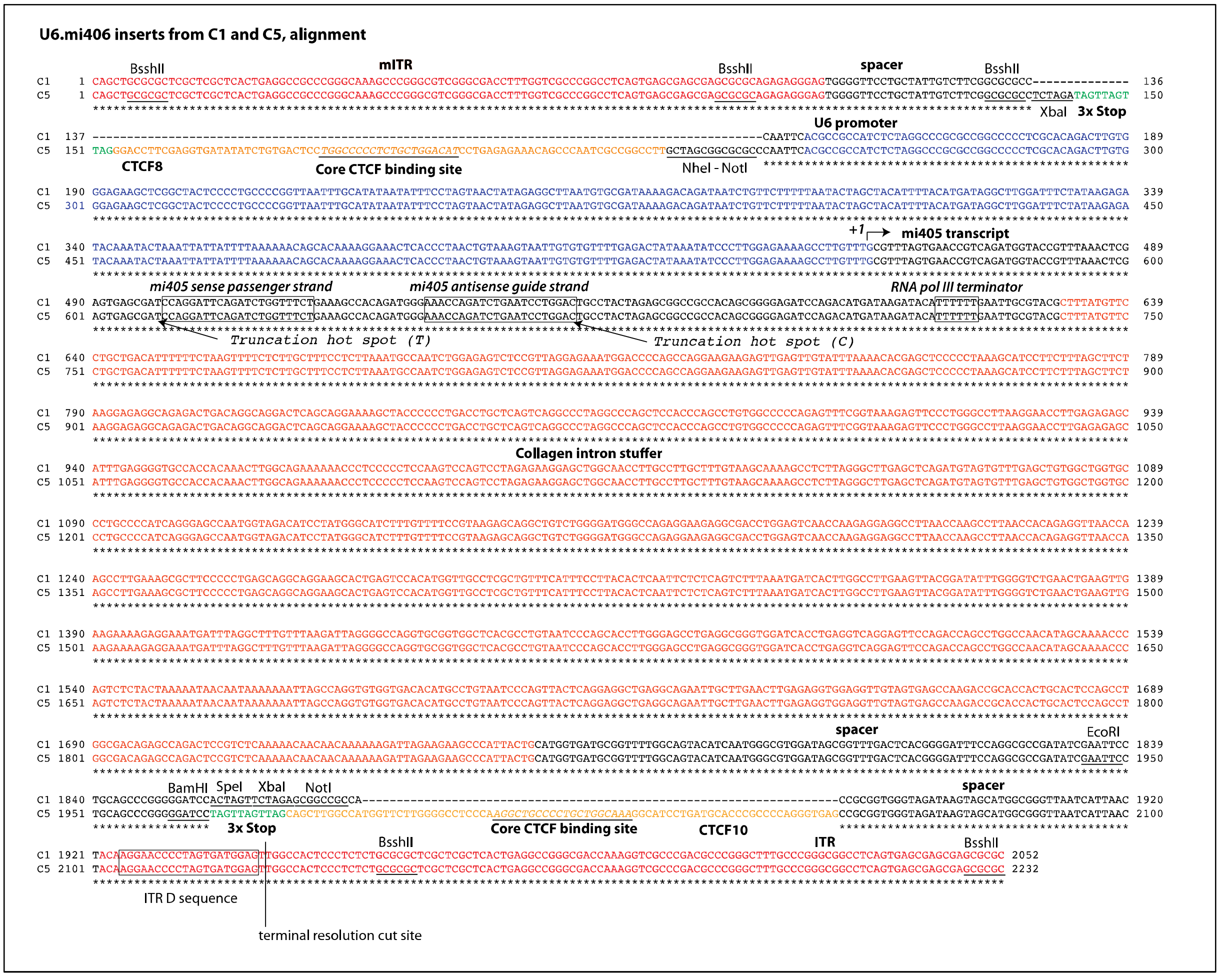

Fig S4. Alignment of U6.mi405 inserts derived from C1 and C5 backbone clones. Relevant features are indicated. The C5 insert is 180 nucleotides larger than the C1 insert due to the addition of two CTCF binding sites and two 3X stop codons.
